# Hypertensive Heart Disease: A Narrative Review Series—Part 2: Macrostructural and Functional Abnormalities

**DOI:** 10.3390/jcm12175723

**Published:** 2023-09-01

**Authors:** Valeriya Nemtsova, Thilo Burkard, Annina S. Vischer

**Affiliations:** 1 Medical Outpatient Department and Hypertension Clinic, ESH Hypertension Centre of Excellence, University Hospital Basel, 4031 Basel, Switzerland; 2 Internal Diseases and Family Medicine Department, Educational and Scientific Medical Institute, National Technical University “Kharkiv Polytechnic Institute”, 61002 Kharkiv, Ukraine; 3 Department of Cardiology, University Hospital Basel, 4031 Basel, Switzerland; 4 Faculty of Medicine, University of Basel, 4056 Basel, Switzerland

**Keywords:** hypertensive heart disease, left atrium remodelling, left ventricular hypertrophy, heart failure, right heart, arrhythmias, hypertension

## Abstract

Hypertensive heart disease (HHD) remains a major global public health concern despite the implementation of new approaches for the management of hypertensive patients. The pathological changes occurring during HHD are complex and involve the development of structural and functional cardiac abnormalities. HHD describes a broad spectrum ranging from uncontrolled hypertension and asymptomatic left ventricular hypertrophy (LVH), either a concentric or an eccentric pattern, to the final development of clinical heart failure. Pressure-overload-induced LVH is recognised as the most important predictor of heart failure and sudden death and is associated with an increased risk of cardiac arrhythmias. Cardiac arrhythmias are considered to be one of the most important comorbidities affecting hypertensive patients. This is the second part of a three-part set of review articles. Here, we focus on the macrostructural and functional abnormalities associated with chronic high pressure, their involvement in HHD pathophysiology, and their role in the progression and prognosis of HHD.

## 1. Introduction

A systematic analysis for the Global Burden of Disease Study 2019 demonstrated that hypertensive heart disease (HHD) showed decreasing trends in the number of disability-adjusted life-years (DALYs) in different age groups between 1990 and 2019 [1]. However, despite the implementation of new approaches for the management of hypertensive patients and the broad availability of effective pharmaceutical agents, HHD remains a growing threat in modern societies and a major global public health concern [2]. It is troubling that the prevalence of hypertension (HTN) is expected to increase to around 1.56 billion by the year 2025, particularly in rapidly developing countries with limited early routine screening protocols [3,4]. The pathological changes occurring in HHD are complex, and alongside many cellular and molecular alterations, involve the development of structural and functional cardiac abnormalities [5]. HHD describes a broad spectrum of changes to the heart ranging from HTN with asymptomatic left ventricular hypertrophy (LVH), either in a concentric or an eccentric pattern, to the final development of overt clinical heart failure (HF) [2,6]. Pressure-overload-induced LVH is recognized as the most important predictor of HF and sudden death and is associated with an increased risk of cardiac arrhythmias [5]. Cardiac arrhythmias are considered to be the most common comorbidity affecting hypertensive patients [3]. In recent years, many studies have focused on the underlying mechanisms linking HTN to arrhythmias, including myocardial ischaemia and left atrial (LA) and left ventricular (LV) enlargement and dysfunction [3].

This is the second part of a three-part set of review articles. The published Part 1 dealt with pathophysiological and microstructural changes in HHD [7]. Here, we focus on the macrostructural and functional abnormalities associated with chronic HTN, their involvement in different HHD presentations, and their role in progression and prognosis.

## 2. Methods

The methods applied for the preparation of the article were published in Part 1 of this review series [7]. In brief: This is a comprehensive systematic review of articles published from 1 January 2000 to 1 January 2022 conducted using the MEDLINE (PubMed), EMBASE, Scopus, Web of Science, and Cochrane Central databases, which were searched using the following keywords and search terms: [hypertensive heart disease], [left ventricular hypertrophy], [adverse cardiac remodelling], [cardiac remodelling], [hypertension], [hypertensive heart failure], [heart failure], [myocardial fibrosis], [hypertensive cardiopathy], [cardiac biomarkers], [circulating biomarkers], [atrial fibrillation], [arrhythmia].

## 3. Review

### 3.1. Left Atrium

#### 3.1.1. Physiological Significance of the Left Atrium

The physiological function of the LA has conventionally been divided into three parts: (a) as a reservoir: the LA collects pulmonary venous return during LV systole and isovolumetric relaxation, (b) as a conduit: the LA passively transports stored blood into the LV during ventricular diastole, (c) as a contractile pump: the LA actively contracts and increases LV filling during the final phase of diastole, providing 15% to 30% of total LV filling [8,9]. Thus, LA function is an important determinant of LV function and cardiac output.

The LA is innervated by the autonomic nervous system (ANS), which modulates LA electrical and contractile activity. The atria receive both sympathetic and parasympathetic innervation, and stimulation or imbalance of either component of the ANS may be associated with increased atrial arrhythmogenicity [10].

It is known that any architectural or structural change in the atrial myocardium can cause significant electrophysiological disturbances. Furthermore, atrial cells respond quickly and comprehensively to various pathological stimuli. In connection with this, it is possible that the atrial myocardium is more sensitive to these stimuli than the ventricular myocardium. Depending on conditions, atrial responses include cardiomyocyte hypertrophy and contractile dysfunction, arrhythmogenic changes, atrial fibroblast proliferation, hyperinnervation, and thrombogenic changes [11]. Experimental evidence supports that hypertensive sympathetic nervous system (SNS) hyperactivity is an early event in both humans and spontaneously hypertensive rats (SHR), initiating the development of both atrial and ventricular remodelling [10,12].

#### 3.1.2. Hypertension-Mediated Left Atrial Remodelling

Microstructural changes in the LA precede macroscopic ones such that an atrium of normal size may already have lost its normal compliance and contractility [13]. HTN may affect the LA via two main interdependent mechanisms: (a) hemodynamic and (b) neurohumoral [10].

Increased afterload of the LV results in higher LV filling pressures, which, in turn, may increase LA pressure, which causes increased atrial wall stress. Due to their thin walls, atria are particularly sensitive to high pressure, which is a trigger for atrial remodelling [10]. LA remodelling is a hallmark feature of HHD and is commonly characterized by LA enlargement and dysfunction. It can be detected using electrocardiography, but echocardiography serves as the gold standard [10].

Neurohumoral activation associated with HTN also induces or contributes to atrial remodelling [10]. In animal models and in the human atrium, major stretch-induced changes in isolated atrial myocytes or the atrial myocardium have been shown to include: (a) an immediate increase in atrial contractile force via the Frank–Starling mechanism, (b) an immediate increase in sarcoplasmic reticulum Ca^2+^ release, and (c) the release of renin–angiotensin–aldosterone system (RAAS) neurohormones, natriuretic peptides and endothelin-1 (ET-1) [10,14,15]. Mechanical stretching of the atrial or ventricular wall in HTN and probably stretch-dependent activation of the RAAS mediators and ET-1 as well as the recruitment of immune cells may also initiate an inflammatory response in the heart [10]. This local inflammation increases fibrosis, promoting further atrial structural remodelling [10]. Sympathetic hyperactivity can be induced by various mechanisms including the activation of neurohumoral mediators [12]. Atrial myocytes in both right and left atria release natriuretic peptides in response to increases in volume or stretch [10]. The consequences of these remodelling processes are LA enlargement, contractile dysfunction, and augmented risk for atrial arrhythmia development [10].

#### 3.1.3. Clinical Significance of Left Atrium Dysfunction

Already in the Framingham Heart Study, LA enlargement was demonstrated to be associated with both the duration of elevated blood pressure (BP) and with the level of systolic BP [16]. The echocardiographic substudy of the LIFE (Losartan Intervention For Endpoint Reduction in Hypertension) study also showed that systolic BP influences LA size in middle-aged and elderly hypertensive patients independently of LVH [17].

However, LA dysfunction in hypertensive patients is not fully understood. Some authors have found that all parts of LA function are affected, whereas others found that only the LA conduit was decreased [18]. Today, changes in LA reservoir, conduit, and booster pump functions can be accurately measured via strain imaging using speckle tracking echocardiography to quantify LA function [19]. A reduction in LA strain reflects the presence of a stiff cavity due to myocardial fibrosis secondary to maladaptive LA remodelling [19]. D’Andrea et al. demonstrated impaired LA myocardial deformation in patients with hypertension compared with age-matched sedentary controls and elite athletes using two-dimensional strain rate imaging to assess LA myocardial function in patients with either physiological or pathological LVH [20]. The authors indicate that the peak systolic myocardial atrial strain was significantly reduced in patients with pathological LVH in comparison to healthy controls and athletes for all atrial segments analysed [20].

Some experimental studies in animal models have shown that there is no change in LA diameter at early stages of HHD [10,21]. However, Lau et al., using the ovine 1K1C model, found an early LA dysfunction while the LV function remained normal, which indicates that macroscopic atrial remodelling may occur before ventricular changes become apparent [21,22]. A significant difference in LA diameter between normotensive and pre-HTN subjects was found in a meta-analysis of echocardiographic studies [23], also suggesting that changes in LA morphology start at very early stages of HHD.

LA remodelling has been shown to be a reliable predictor of adverse cardiovascular (CV) outcomes: new-onset HF, atrial fibrillation (AF), stroke, overall mortality after myocardial infarction (MI), and all-cause mortality [8,9,21]. In the echocardiographic substudy of the LIFE study, LA diameter/height was shown to predict subsequent CV events in hypertensive patients with LVH before starting antihypertensive therapy, independently of other clinical risk factors [24]. Kaminski et al. demonstrated that not only is increased LA size a marker of LV diastolic dysfunction that has been shown to be predictive of adverse CV outcomes, but also that in hypertensive patients, a reduced contribution of LA contractile function to ventricular filling during diastole is also a strong predictor of adverse cardiac events and death [25]. The WARCEF (Warfarin versus Aspirin in Reduced Ejection Fraction) trial, which performed two-dimensional echocardiography with LA volume index (LAVI) measurement in patients with systolic HF in sinus rhythm (approximately 60% of patients were hypertensive), showed that moderate or severe LA enlargement is significantly associated with death and HF hospitalization despite treatment with the at this time recommended HF regimen, including antithrombotic medications [19,26]. Similarly, in patients with HF, including HF of hypertensive origin, decreasing left atrial emptying function (LAEF), as measured by cardiac magnetic resonance (CMR) imaging, was associated with impairment of different cardiac function measurements like left ventricular ejection fraction (LVEF) or right ventricular ejection fraction (RVEF), higher circulating biomarkers like circulating N-terminal pro-brain natriuretic peptide (NT-proBNP), and worse prognosis in terms of survival [27,28]. LA strain has also been shown to predict death and hospitalisation independently of the degree of LV remodelling or dysfunction [19]. However, LA strain has its limitations. These are mainly related to the lack of consensus on which regions to include in the calculation of global strain, the need for the standardisation of equipment and analytical techniques, and, finally, reliable outcome data from large prospective trials to confirm the additive predictive ability of LA function. These issues need to be addressed before these measurements can be widely accepted [19,29].

On the other hand, the treatment of hypertension is associated with a reduction in LA enlargement [30]. Interestingly, Parikh and colleagues have shown in SHR hearts that atrial myocyte hypertrophy (and fibrosis) may be reversed by exogenous administration of the pleiotropic hormone Relaxin, even in the late stages of chronic HTN [10,31]. In the same study, the authors showed a suppressive effect of 2 weeks of systemic Relaxin administration on AF inducibility in SHR hearts [31]. The results of this study could be the background for a new approach for the development of more effective therapies for AF in hypertensive patients.

However, although the role of atrial function in heart disease, particularly HHD, is of great clinical relevance, the exact mechanisms of initial and progressive structural and functional dysfunction are not well understood. The role of neurohumoral factors in LA remodelling, in particular RAAS or pro-inflammatory pathways, is currently extensively studied. Ongoing and future studies will improve our understanding of the atrial remodelling dynamics in HHD, enabling the development of more effective strategies for the treatment and prevention of adverse CV events in these patients.

### 3.2. Left Ventricular Hypertrophy

#### 3.2.1. Left Ventricular Hypertrophy Classification and Patterns

The LV remodels over the life course as an adaptive response to aging, exposure to CV risk factors, and myocardial injury [32]. LVH is defined as an increase in LV wall thickness or mass quantified by measurements of postmortem weight, by electrocardiographic (ECG) criteria, and by echocardiography or CMR [33,34]. The prevalence of LVH in HHD varies according to the characteristics of the population studied, the applied methods (ECG, echocardiography), and criteria. In early works, the prevalence of LVH among hypertensive patients was 23% to 50% and 0% to 10% in normal subjects [35,36]. Later, Cuspidi and colleagues indicated a prevalence of LVH ranging from 36% to 41% (depending on the criteria applied) in a pooled hypertensive population of 37,700 untreated and treated patients [37].

There are three main patterns of LVH, first described by Ganau et al.: (a) concentric hypertrophy, (b) eccentric hypertrophy, and (c) concentric remodelling [38]. Concentric and eccentric LVH are the two main patterns of LVH. The American Society of Echocardiography/European Association of Cardiovascular Imaging (ASE/EACVI) proposed LV mass index (LVMi) and relative wall thickness (RWT, ratio of LV posterior wall thickness, and LV internal dimensions) as classifying variables for concentric and eccentric LVH, using RWT of 0.42 and LV mass/Body Surface Area (BSA) as LVMi of 95 g/m^2^ in female and 115 g/m^2^ in male as the determining cut-off values [39]. The reference upper limits of normal LV mass according to 2D echocardiographic measurements are 88 g/m^2^ in women and 102 g/m^2^ in men with 2D echocardiographic imaging [40]. Alternatively, the evaluation of LVH with indices based on height alone has been reported [41]. Studies suggest that indexing to height raised to allometric powers such as 1.7, 2.13, and 2.7 has advantages over indexing to BSA, especially when estimating the prevalence of LVH and attempting to predict events in overweight and obese individuals [40]. Indexation to height^2.7^ has been proposed to be similarly sensitive in detecting obesity-related and -unrelated LVH, and LVH has been defined as LVMI ≥ 49 g/m^2.7^ in men and ≥45 g/m^2.7^ in women [40,41,42]. However, despite the fact that most large-population studies using LV mass were indexed to BSA, the use of height, weight, or BSA as an indexing term remains controversial.

Based on LVMi and RWT measurements, four geometric patterns of LV remodelling can be allocated: normal geometry (LVMi and RWT are normal), concentric remodelling (increased RWT but normal LVMi), concentric hypertrophy (LVMi and RWT are increased), and eccentric hypertrophy (increased LVMi with normal RWT) (Table 1) [8,32,39].

Echocardiographic studies have demonstrated that hypertensive patients can have any of these LV geometry patterns [43].

An alternative and widely discussed four-group LVH classification initially proposed by Gaasch and Zile is based on LV mass, end-diastolic volume (EDV), and RWT [44]. Depending on EDV dilatation, this classification subdivides both eccentric LVH and concentric LVH into two sub-groups (see Table 2) [39,44,45]. However, further studies are required to clarify an incremental value of this classification for risk stratification and therapeutic implications in patients with HHD.

Concentric LVH is more common in middle-aged and elderly patients, is associated with lower cardiac output, and predicts poor prognosis [46]. Eccentric LVH is more common in younger patients and is associated with higher cardiac output [47]. It is not fully understood why patients develop a specific LVH pattern in response to HTN.

It should be noted that differences exist between the patterns of LVH in patients with HTN when compared with physiological adaptations, mainly observed in athletes, independently of age and sex in adult athletes, or pregnant women [48]. This probably can be explained by the presence of myofiber disarray, impairment in active myocyte relaxation, and extracellular fibrosis in HHD [49]. In physiological hypertrophy, an increase in active muscular mass and normal myofiber architecture but only minimal or no fibrosis has been observed. Furthermore, the systolic and diastolic functions are usually normal [50,51].

In recent years, special attention has been paid to focal hypertrophy as an early finding in LV geometric remodelling in hypertensive patients. It has been shown that the septal base is thicker than the mid apical part in mild and moderate HTN [52]. Basal septal hypertrophy may be the first region affected in an earlier stage of HHD than LVH [53] and could result in a greater degree of tissue dysfunction in the basal septum compared to the LV free wall in advanced hypertensive disease [52,53]. However, further research is needed to accurately determine the diagnostic value of basal septal hypertrophy in HHD, as early detection of the cardiac remodelling and cardiac target organ damage in HTN may be of great importance in identifying patients requiring intensive BP monitoring and treatment.

#### 3.2.2. Clinical Significance of Hypertensive Left Ventricular Hypertrophy

The fundamental effect of chronic pressure and volume overload on LVH development is well known. In particular, night-time BP examined using ambulatory blood pressure monitoring (ABPM) was identified as the main independent predictor of LVH and altered geometry [54]. Except for pressure and volume overload, factors such as demography (ethnicity, gender), obesity, the activity of RAAS, and genetics may significantly influence the development of LVH [5,36,47,55]. Animal and human studies provide persuasive evidence for a significant genetic contribution to the total variance in cardiac mass [56]. It has also been shown that in the general population, LVH is common and thus present in 16% of European Americans and up to 43% of African Americans [57]. Similar results have been obtained in studies or substudies specifically designed with focus on ethnicity, showing that regardless of the method used to diagnose LVH (ECG, echo or magnetic resonance imaging (MRI)), Black populations (including African Americans) have a two- to three-fold higher prevalence of LVH than the general population, and they develop a greater degree of concentric LV remodelling or hypertrophy for a given degree of hypertension in comparison to their white counterparts [57,58]. Moreover, Lewis et al. demonstrated that the prevalence of malignant LVH, as characterised by minimal elevations in cardiac biomarkers at high risk for developing HF, was three-fold higher in Black men and women than in white men and women, resulting in a higher incidence of HF in Black men and women [59].

#### 3.2.3. Left Ventricular Hypertrophy as a Risk Factor for Cardiovascular Events

Early studies on animal models with ascending aortic constriction and in patients with aortic stenosis in the 1970s led to the concept that LVH is a compensatory mechanism in pressure overload to reduce wall stress, thereby allowing for the preservation of LVEF [43]. However, later, these data were not consistent with subsequent epidemiological studies showing that LVH was associated with adverse clinical outcomes [43]. Today there is good evidence that increased LV mass alone can predict adverse outcomes, and it is known that the risk of CV mortality increases with increasing LV mass [36,60]. Numerous prospective epidemiological studies including the well-known Framingham Heart Study convincingly demonstrated that LVH is a major CV risk factor and is strongly associated with an increased risk of CV events that is independent of arterial pressure levels, including a 4.5-fold increased risk for sudden cardiac death [46,61,62,63]. Recent data also demonstrated that LVH is among the leading causes of death worldwide, and that significant LVH increases the risk of sudden cardiac death six- to eight-fold in men and three-fold in women [64,65]. In addition, the Framingham Heart Study demonstrated that definite electrocardiographic evidence of LVH was associated with six-fold and eight-fold increases in coronary heart disease (CHD) and CV mortality risk, respectively [46]. A series from the Bronx Longitudinal Aging Study showed that in a cohort of men and women aged 75 to 85 years at baseline, there was a significantly increased mortality rate for subjects with compared to subjects without baseline ECG LVH [66]. Several studies confirmed that persistent LVH is an independent predictor of all-cause mortality [66,67]. However, taking into consideration the low sensitivity and specificity of LVH by ECG criteria, particularly in young male patients, the true risk of major CV events in studies using ECG may be biased [34]. The echocardiographic assessment of LVH has a higher specificity and sensitivity (both ≥80%) than ECG [36]. Moreover, in general and according to studies in cohorts of patients with essential hypertension, both concentric and eccentric LVH are associated with a higher risk of non-fatal or fatal CV events and all-cause mortality, particularly patients with concentric LVH [46,54,67,68]. There is increasing evidence that the use of MRI allows for the identification of subclinical changes in LV size and systolic function that are difficult to detect using other available non-invasive methods such as echocardiography [33,69]. In addition, MRI can be especially helpful in differentiating hypertrophic cardiomyopathy from HHD [70].

#### 3.2.4. Left Ventricular Hypertrophy and Arrhythmias

The presence of LVH has been reported to be associated with supraventricular (SV) and ventricular (VA) arrhythmias. A meta-analysis that included predominantly hypertensives showed a significantly higher incidence of both supraventricular as well as ventricular arrhythmias in patients with LVH in comparison to patients without LVH [71]. The authors of this meta-analysis also demonstrated a 3.4-fold greater risk of SV arrhythmias and 2.8-fold greater risk of VA in patients with LVH [71].

LVH is also well known to be a predictor of AF in the hypertensive population [72]. In untreated hypertensive subjects who were in sinus rhythm at baseline, it was found that age and LV mass were the only independent predictors of AF [72,73]. The reason for increased arrhythmogenicity in LVH is not clearly understood. Factors such as myocardial ischemia, scar tissue, neuroendocrine factors, ventricular wall stress, and electrolyte disturbances are thought to enhance the proarrhythmic potential of LVH [71].

#### 3.2.5. Left Ventricular Hypertrophy and Heart Failure

There is considerable evidence that LVH is an intermediate stage in the progression to HF. A broad range of molecular pathways, as well as oxidative stress, inflammation, myocardial fibrotic process, and aggravated diastolic stiffness, are thought to be involved in the progression of LV hypertrophy leading to the development of HF [74]. In the initial phase, termed the “adaptive phase” or “compensatory phase”, concentric hypertrophy apparently represents the result of an attempt by the LV to maintain wall stress in response to chronic pressure and volume overload to preserve the cardiac output [5]. It is mainly associated with an increase in cardiomyocyte size and cardiac mass. Adaptive hypertrophy can transit to maladaptive remodelling with severe cardiomyocyte loss and fibrotic remodelling, which leads to stiffening of the ventricular wall, resulting in impairment of LV contraction and relaxation, disturbance of electrical conduction, and reduced capillary density [47]. This stage is associated with reduced ejection fraction (EF) and LV dilatation, with systolic and diastolic dysfunction, and predisposes the development of HF, arrhythmias, myocardial ischemia, or even sudden death [5,47].

However, it is still not fully clarified why not all patients with hypertension develop LVH, and why certain patients develop a specific pattern of LVH as a response to hypertension. At the same time, despite controlling for known HF risk factors, there is an unclear understanding of the pathways that lead to a progressive course of LVH in some individuals but an uncomplicated course in many others.

#### 3.2.6. Left Ventricular Hypertrophy as Therapeutic Target

Several studies have shown the regression of LVH with antihypertensive treatment, which reduces, but does not entirely eliminate the risk of stroke, myocardial infarction, and all-cause mortality [43,47]. Accordingly, the LIFE study showed that antihypertensive therapy was associated with a reduction in the electrocardiographic voltage criteria of LVH, presented on a ECG screening, and a subsequent reduced likelihood of CV morbidity and mortality, independent of treatment modality and BP lowering [75]. Despite the lack of adjustment for other clinical variables in the outcome analyses, the data from the HOPE (Heart Outcomes Prevention Evaluation) trial provided the strongest evidence that ramipril-based antihypertensive therapy was also associated with the regression of electrocardiographic LVH and improved prognosis by reducing the risk of death, MI, stroke, and HF [76]. A meta-analysis indicated that regression of echocardiographic hypertensive LVH is associated with a reduction in CV events, even after adjustment for various confounders [77].

The beneficial effects of RAAS blockers on cardiac and electrophysiological LV remodelling appear to be independent of reduction in BP [63,78]. Overall, there is ample evidence that angiotensin-converting enzyme (ACE) inhibitors, angiotensin II receptor blockers (ARBs), and calcium channel blockers (CCBs) are more effective than beta blockers (BBs) and diuretics in reducing LV mass [63,79,80]. Mineralocorticosteroid receptor antagonists, although not first-line antihypertensive drugs, have been shown to be as effective as ACE inhibitors in LVH regression [81]. The results of a randomized, double-blind, active-controlled trial showed greater reductions in LV mass in the group of sacubitril/valsartan (S/V) than in the olmesartan group in patients with HTN and elevated pulse pressure [82]. In addition, the authors also found that the decrease in LV mass index might be to some extent independent of SBP. Of note, large intervention trials have shown that a combination of two or more first-line antihypertensive medications not only provides more satisfactory BP control but also more effective CV protection, especially in hypertensive patients with one or more CV risk factors beyond HTN [83,84]. For example, the PICXEL (Perindopril/Indapamide in a double-blind Controlled study versus Enalapril in Left ventricular hypertrophy) and REASON (pREterax in regression of Arterial Stiffness in a contrOlled double-bliNd) trials showed that combination therapy with an ACE inhibitor/diuretic resulted in a significantly greater pronounced decrease in LV mass/hypertrophy than monotherapy with either an ACE inhibitor or a beta-blocker in hypertensives [85,86]. Through improvement of adherence, single-pill combinations can even further enhance the BP-lowering effect of the substances, and by this potentially influence the outcome of HHD [87,88,89,90]. Current guidelines for hypertension management clearly support greater use of single-pill combination therapy, especially in high-risk patients [2,84].

However, future studies are needed to evaluate whether LVH regression is beneficial in all groups of hypertensive patients. There is experimental evidence that LVH regression may reduce the risk of cardiac events by improving coronary flow reserve, reducing the risk and incidence of cardiac arrhythmias, reversing systolic and diastolic dysfunction, and being associated with less progression or regression of atherosclerosis, thus explaining the reduction in both cardiac and cerebrovascular events [77,91]. Nevertheless, the mechanisms by which LVH regression translates into better CV outcomes in HTN remain largely uncertain [90].

### 3.3. Heart Failure

#### 3.3.1. Heart Failure Definition and Classification

According to a new universal definition, HF is a clinical syndrome, and it continues to have a high prevalence and a high risk of death, especially among older adults [92,93,94]. HF has been historically understood as being caused primarily by LV dysfunction. New data obtained over the last years have contributed not only to the optimization of clinical classification of HF by stages (from stage A to D) but revision of the classification of HF according to left ventricular ejection fraction (LVEF). This new classification includes HF with reduced LVEF (HFrEF): HF with an LVEF of ≤40%; HF with mildly reduced EF (HFmrEF): HF with an LVEF of 41% to 49%; HF with preserved EF (HFpEF): HF with an LVEF of ≥50% [93,94]. Moreover, Bozkurt et al. proposed the allocation of a HF with improved EF (HFimpEF): HF with a baseline LVEF of ≤40%, a ≥10-point increase from baseline LVEF, and a second measurement of LVEF of >40%; but this need further investigations [93].

#### 3.3.2. Heart Failure and Hypertensive Heart Disease

Cardinal clinical manifestation of HF due to HHD is the presence of LV hypertrophy in the absence of any other cause except arterial hypertension [95]. Once LVH develops, the risk of developing both HFpEF and HFrEF increases dramatically, even when controlling for known HF risk factors [49]. In large HF registries and studies, HTN was present in 70–91% of patients with HF overall [49,96]. In the ESC Heart Failure Long-Term Registry of ambulatory patients with HF, treated HTN was present in 56% with HFrEF, 60% with HFmrEF, and 67% with HFpEF, respectively [97,98]. Interestingly, in a real-world registry from Europe, HTN was identified as the underlying cause in 30% of patients with HFmrEF, compared with 16% in HFrEF and 49% in HFpEF [98]. HTN has been found to precede the development of HF by an average of 14 years [99]. Therefore, the American College of Cardiology (ACC) and the American Heart Association (AHA) recommend considering HTN as a risk factor of HF even in the absence of detectable structural heart damage [100]. Notably, the association between BP and cardiovascular diseases was indicated to be stronger in the Asian than in the Western population [101]. A high prevalence of heart failure as a complication of hypertension is one of the hallmarks of hypertension in Asia [101].

The progression from HTN to HF is complex and multifactorial. In the most widely accepted model of hypertensive HF, progressive hypertrophy and fibrotic changes in the heart lead to progressive diastolic dysfunction, ultimately leading to elevated left-sided filling pressures and diastolic HF [7,49]. Fibrosis may contribute to the pathophysiological changes of HHD through diverse pathways [7]. Initially, the accumulation of collagen fibers contributes to impaired diastolic function. High LV myocardial diastolic stiffness further impairs diastolic filling and increases myocardial passive stiffness in these patients, leading to compromised cardiomyocyte contraction and myocardial force, thus impairing systolic performance [102,103]. Differences in the pressure load (which may not be discernible by office blood pressure measurements) may be associated with the considerable variability in hypertensive patients progressing to HHD [43]. Therefore, patients with a non-dipping status during the night have a more pronounced impairment of LV diastolic and systolic function along with increased peripheral arterial stiffness compared with those with a normal nocturnal dipping pattern [104]. Importantly, a non-dipping pattern is an independent risk factor for impaired systolic LV function in patients with HTN [104]. Concomitant medical conditions, underlying neurohumoral status, and genetic predisposition are also considered to predispose the progression of HTN [43,105]. As a result, in the presence of chronic volume and pressure overload, a subset of patients progresses to systolic dysfunction and then to systolic HF [49].

Based on the pathophysiologic and clinical impact of hypertension on the heart, Messerli and colleagues described four stages of HHD: (1) hypertension without LVH (only isolated diastolic dysfunction), (2) LV diastolic dysfunction with concentric LVH (asymptomatic hypertension), (3) symptomatic HFpEF, and (4) dilated cardiomyopathy with HFrEF [49,106].

Diastolic dysfunction is thought to be one of the first discernible manifestations of left ventricular disease in HTN. On the other hand, HTN is the major modifiable risk factor for the development and progression of HFpEF and is thought to be the leading aetiology for HFpEF development [6,106,107].

Importantly, the prevalence of HFpEF has been increasing over the last decade and by now, HFpEF is the most common form of HF, exceeding the prevalence of HFrEF by 10%. It is becoming a growing public health problem with substantial morbidity and mortality that currently affects 9% of people older than 60 years and accounts for approximately half of all clinical HF presentations [60,103,108]. This is primarily owing to the increasing age of the population and thus increasing prevalence of conditions that predispose HF development, particularly HTN [109]. The importance of diastolic HF in HHD is underscored by the fact that the outcome and survival of patients with a HFpEF appear to be similar to those of patients with a HFrEF [110], and the quality of life of HFpEF patients is as poor as in HFrEF patients [103,110].

**Figure 1:**
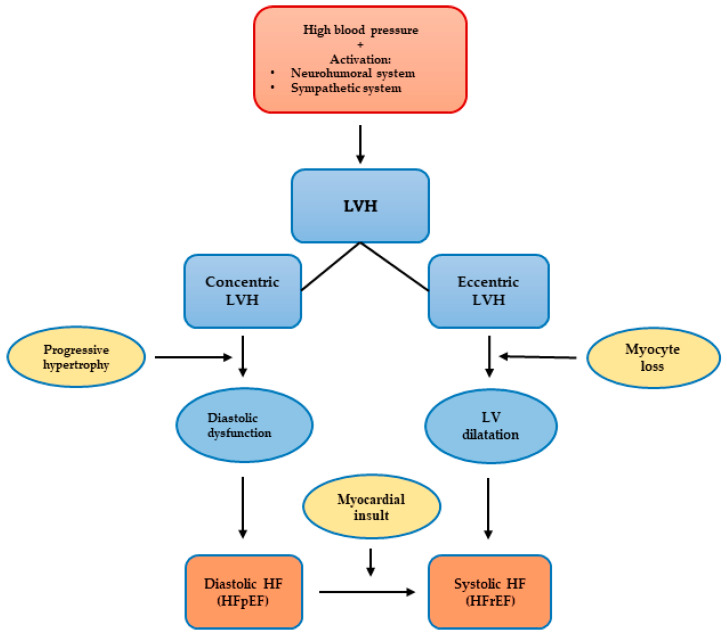
Proposed mechanism for the progression of hypertensive heart disease to heart failure patterns, LVH: left ventricular hypertrophy, LV: left ventricular, HFpEF: heart failure with preserved left ventricular ejection fraction, HFrEF: heart failure with reduced left ventricular ejection fraction.

Compared to those who develop HFpEF, clinical HFrEF develops generally through disproportionate myocyte loss rather than hypertrophy (Figure 1) [49].

Those with eccentric hypertrophy are more likely to develop HFrEF than those with concentric hypertrophy, and at the same time, there are data that there may be a direct progression to HFrEF without concentric hypertrophy [43,49]. It is suggested that apoptosis, hyperactivity of RAAS, oxidative stress, and ischemia may play an important role in the transition from compensated LVH to HF in the presence of chronic pressure overload, although most of the underlying mechanisms are still unclear [110]. Despite significant advances in the therapy of systolic HF, once HFrEF develops in patients with HHD, the prognosis becomes markedly worse. Slivnick and colleagues provided data about the estimated 4-year survival rate, which is about 60% for patients with symptomatic New York Heart Association (NYHA) class II-III HF [49].

Patients with HFmrEF have, on average, features that are more similar to HFrEF than HFpEF, and are less likely to have AF and non-cardiac comorbidities [94]. However, ambulatory patients with HFmrEF have a lower mortality than those with HFrEF [94]. The true prevalence of HFmrEF and its impact on the course and prognosis in HHD remains to be determined.

In hypertensive humans, sustained pressure overload leads to the progression of diastolic dysfunction, decompensation of LV concentric hypertrophy, and hypertensive HFpEF development. In contrast, sustained volume overload leads to LV dilatation progression, decompensation of eccentric remodelled LV, and HFrEF development [106,110]. In animal models of pressure overload, it has been demonstrated that prevention of LVH by inhibition of calcineurin either pharmacologically with cyclosporine or via transgenic overexpression of an endogenous inhibitor of calcineurin myocyte-enriched calcineurin-interacting protein-1 (MCIP1) did not lead to ventricular dilation or decline in fractional shortening [43,111,112]. In other transgenic mouse models, the prevention of concentric hypertrophy also was not associated with ventricular dilatation, and dilated cardiac failure did not develop [43]. These data suggest that inhibition of the development of concentric LVH could be a potential therapeutic target in pressure-overload conditions, including HHD, which needs to be scrutinized in humans [43]. Therefore, Velagaleti et al. showed over a mean follow-up of 21 years that participants with eccentric hypertrophy had a higher propensity for the development of HFrEF than those with concentric hypertrophy, while those with concentric hypertrophy were more prone to HFpEF [113]. At the same time, there are data that there may be a direct progression to HFrEF without concentric hypertrophy [43,49]. Recent advances clearly show that despite the similar clinical presentation of HFrEF and HFpEF, these syndromes are mechanistically distinct pathophysiological entities, based on gene expression analyses and response to neurohormonal therapies [108]. Consequently, a transition from HFpEF to HFrEF in patients is rare [108]. However, HFpEF is not the same as LVH, as in some randomised controlled trials (e.g., I-PRESERVE, CHARM-Preserved, TOPCAT) approximately 1/3 to 2/3rds of patients with HFpEF did not meet LVH criteria [108].

Borlaug and Redfield named conditions predisposing accelerated myocyte dysfunction on the background of hypertension a “second hit” [114]. Such a second hit may occur from MI, some medications, toxins, or genetic polymorphisms. Hypertensive patients with concentric LVH who experience a myocardial infarction seem to be at increased risk for developing dilatation and cardiac failure [43,115]. In hypertensive patients with concentric LVH and a normal LVEF at baseline, it was shown that patients who developed a reduced EF were more likely to have had a MI during the follow-up period than those without progression to reduced EF (41% vs 8%, respectively) [115]. The authors of the Cardiovascular Health Study also suggest that concentric hypertrophy does not commonly progress to dilated cardiac failure after 5 to 7 years of follow-up in the absence of interval MI [43,116]. Ischemia is by far the most common “second hit”, often instigating the progression from HFpEF to HFrEF; however, it should be noted that, reversely, large observational studies have shown that only nearly half of hypertensive patients who progressed to systolic dysfunction had a history of previous MI [49,100].

It is important to remember that a significant portion of HFpEF patients do not have evidence of LVH on echocardiogram or have apparently normal measurements of diastolic function, but conversely, many older adults have LVH and/or LV diastolic dysfunction but do not have clinical evidence of HFpEF [107]. This suggests that the mechanism of hypertensive HFpEF is complex and heterogeneous with multiple contributors to its pathophysiology. A large number of circulating biomarkers have been proposed to improve the diagnosis and management of HF patients, but very few of these biomarkers have entered into clinical practice [95]. Therefore, the combination of LVH with increased levels of high-sensitivity cardiac troponin T and NT-proBNP is recommended for the identification of patients at highest risk for developing symptomatic HF, especially HFrEF [95].

One additional factor that has to be kept in mind is that chronic hypertension and HFpEF are often accompanied by renal dysfunction, and this combination is associated with greater LVH and worse cardiac haemodynamics, leading to RV dysfunction and worse prognosis [108]. Also, metabolic, endocrine, and humoral signalling between the heart and the adipose tissue has been proposed to play an important role in the pathophysiology of HFpEF [108].

In advanced HF, systolic BP is usually low, even in previously hypertensive patients. This phenomenon is termed “decapitated hypertension” and is used to describe the decrease in BP resulting from reduced systolic function and fall in cardiac output despite the presence of compensatory mechanisms such as peripheral vasoconstriction, contributing to the progression of HF [106].

#### 3.3.3. First-Line Antihypertensive Drugs and Heart Failure in the Setting of Hypertensive Heart Disease

Based on the current evidence, the guidelines recommend the treatment of HTN in patients with HF with an initial combination of ACE inhibitors or ARBs, beta-blockers, and a thiazide/thiazide-like diuretic, alternatively a loop-diuretic [2]. If HTN is still uncontrolled, they recommend adding mineralocorticoid receptor antagonists [2]. RAAS inhibitors and CCBs have been shown to reverse LVH remodelling better than BBs and diuretics. However, trials have demonstrated that CCBs provide less protection against HF than other first-line antihypertensive drugs [63]. Therefore, in the CONVINCE (Controlled Onset Verapamil Investigation of Cardiovascular End Points) trial, the incidence of HF was 30% higher in patients treated with verapamil than in those treated with a diuretic [117]. Meta-analyses have shown an increased risk of HF with the use of intermediate-acting CCBs from the dihydropyridine subgroup compared with the main alternative antihypertensive therapy, including β-blockers, diuretics, ACE inhibitors, or ARBs [118]. Amlodipine therapy was associated with a 25% higher risk of HF [118]. The use of CCBs in combination with ACE or β-blockers also does not seem to reduce the risk of heart failure in HTN [118]. It has been suggested that the effect of dihydropyridine CCBs on HF is a BP-independent class effect resulting primarily from cardiomyocyte apoptosis and inhibition of intracellular calcium flux [118].

In general, the influence of first-line antihypertensive drugs on the risk of HF development and on the course of HF in hypertensive patients is under great attention in appropriate guidelines and the consensus is that the most important task is to treat blood pressure to the recommended targets by the use of first line antihypertensive treatments [2,6,94]. However, to date, whether these groups have similar therapeutic and prognostic effects in different types of hypertensive HF remains largely unclear and requires further research especially in the era of initial combination therapies [2].

### 3.4. Right Heart (Right Atrium and Ventricle)

The right ventricle (RV) and right atrium (RA) have long been considered unessential in patients with HTN. In recent years, with the arrival of new reliable and reproducible techniques such as speckle tracking imaging, 3D echocardiography, and cardiac magnetic resonance (CMR), the ability to image and assess various aspects of the RV and RA in hypertensive patients has increased significantly.

#### 3.4.1. Involvement of Right Heart in Hypertensive Heart Disease

Recent studies have revealed functional and mechanical changes as well as structural remodelling of the right heart in patients with HTN that have not been previously associated with HTN [119]. Thus, RV wall thickness was found to be significantly greater in patients with HTN than in those without HTN [119]. RA dilatation and dysfunction were found to be significant contributors to RV diastolic dysfunction due to its relationship with RV filling pressure, which is important in the biventricular diastolic dysfunction often seen in hypertensive patients [119].

Based on the results of their study, Zhang et al. suggested that regional diastolic dysfunction in patients with primary HTN may occur first in the RV before the LV [120], which was previously shown in experimental animal models [121].

Liu and colleagues underlined three mechanisms that may be responsible for structural remodelling of the RV in HTN and may explain the pathophysiological processes linking the right heart and the development and progressing of HHD [122]. First, the incapability of sustaining long-term pressure overload, together with overstimulation of the sympathetic and RAAS system, results in the promotion of fibrosis and alterations in the RV extracellular matrix in hypertension [7,19,122]. The role of the RAAS has also been supported by data from another study that showed an improvement in RV global function independently of BP reduction in patients with mild HTN treated with RAAS inhibitors [123]. Second, as hypertension-induced LVH as well as pressure- or volume-overload-stimulated RV dilation commonly affect the interventricular septum, this could potentially affect RV function through left-right ventricular interactions [19,122]. The influence on RV function may be exerted not only by the interventricular septum, but also by the circular and spiral bundles of muscle fibres that encircle both ventricles [121]. Further, studies in isolated hearts suggest that the LV contributes to 65% of the work of the normal RV [124]. And third, elevated LV end-diastolic pressure as a result of LV systolic or diastolic dysfunction may lead to pulmonary venous hypertension and consequently high pulmonary arterial systolic pressure, culminating in RV dysfunction [122].

A cross-sectional study investigating the influence of different LV geometric patterns via 2D and 3D echocardiography reported that the RA conduit and reservoir function gradually decreased and that the RA pump function gradually increased from hypertensive patients with normal LV geometry to those with LV dilatation and concentric LVH [119,121]. It has also been revealed that the RV endocardial layer is more impacted by HTN than the epicardial layer in hypertensive patients [125].

#### 3.4.2. Right Heart Adaptions as a Predictor of Cardiovascular Outcomes

New approaches have shown that RA and RV dilatation and dysfunction provide the background for electrical instability with the onset and recurrence of AF, which is especially frequent among hypertensive patients, and loss of contractile force, leading to the right HF [19,126]. The association of severe LV and RV failure is often found in patients with end-stage HF who have limited treatment options [19,127]. Thus, secondary to a failing LV, RV dysfunction also contributes to the development of the HF syndrome and worsening of symptoms.

Previous studies have suggested that subclinical RV dysfunction may be observed early in the course of HTN [122,128]. Tadic and colleagues showed in a longitudinal study a correlation of RV hypertrophy, RV systolic dysfunction, RV diastolic dysfunction, and RA dilatation with adverse CV outcomes (AF, MI, myocardial revascularization, HF, stroke, or CV death) in the hypertensive population during a 9-year follow-up [119,129]. However, after adjustment for demographic and clinical parameters, only RV hypertrophy remained an independent predictor of these events [119,129]. Other studies have shown that RA dilatation and/or dysfunction are significant predictors of CV morbidity and mortality in hypertensive patients [121,130,131]. The Multi-Ethnic Study of Atherosclerosis (MESA), a multicentre prospective cohort study designed to investigate the prevalence, correlation, and progression of subclinical CV disease in a multi-ethnic population free of clinical CV disease at baseline, showed that RV hypertrophy as assessed by CMR was associated with a more than threefold increased risk of HF or death compared with those with normal RV mass [132]. In contrast, in experimental RV failure, RV hypertrophy was not clearly shown to be a strong predictor of outcome [124].

Taken together, new imaging modalities have revealed significant RV and RA changes in patients with HTN. The causes of right heart adaptions seen in essential HTN are complex and remain largely unclear. At first sight, these adaptions seem subtle and subclinical, but they are associated with adverse outcomes in hypertensive patients and should therefore be included in the clinical assessment protocols of patients with HTN [119]. Also, early detection of RA and RV enlargement and dysfunction can predict patients at higher risk of arrhythmias, especially the development of AF, and allow for their regular monitoring. However, simultaneous RV and LV involvement needs to be scrutinized in further studies.

#### 3.4.3. First-Line Antihypertensive Drugs and Right Heart in the Setting of Hypertensive Heart Disease

The involvement of the RV and RA in the pathogenesis of HHD and their influence on the clinical pattern and prognosis of HHD have become a point of special interest only in the last decade. This may partly explain the lack of randomised controlled trials designed to evaluate the influence of different groups of antihypertensive drugs on right heart function in HHD patients. A beneficial effect of RAAS inhibitors on RV function has been demonstrated in a few studies [123]. The meta-analysis of observational studies on the effect of S/V on RV function showed a significant improvement in RV function and pulmonary circulation in patients with HFrEF, but the mechanisms by which S/V improves RV function are not fully understood [133].

Even though it seems to make sense that β-blockade should have a therapeutic benefit in the failing RV, there is no evidence of the benefit of β-blockers in RV failure on the background of HTN. Moreover, the results in adults with RV failure are controversial [124,134,135].

Further controlled trials are needed to better elucidate the influence and mechanisms of antihypertensive treatment effects on the right heart in HHD patients.

### 3.5. Arrhythmias and Conductions Disturbances

HHD is associated with different cardiac supraventricular and ventricular arrhythmias, most commonly AF, especially in those with LVH or HF.

LA dilatation, a hallmark of atrial structural remodelling, was non-invasively recorded via ECG over decades until echocardiography took over as the standard tool to evaluate left atrial size and nowadays also function. As early as 1963, G. Ross demonstrated the increase in P-wave duration and amplitude in hypertensive patients and the correlation of P-wave characteristics with different forms of HTN [10,136]. Early ECG recordings from SHR showed a progressive, age-associated increase in the P-R interval and QRS duration, suggesting impaired atrioventricular conduction, but prolongation of the P-R interval was not observed in hypertensive patients [10,136,137].

#### 3.5.1. Pathophysiological Substrate for Arrhythmogenic and Conduction Disturbances in Hypertensive Heart Disease

It is considered that several different mechanisms contribute to the pathophysiology of arrhythmias in HHD.

Elevated systemic pressure influences the size and function of the LA, which makes uncontrolled HTN a key risk factor predisposing the development of AF [138,139,140]. Studies in animal models also suggest that considerable remodelling of ion currents in atrial myocytes may affect intracellular ion handling (Na^+^, Ca^2+^) and propensity for arrhythmia in HHD [10,141]. Remodelling of atrial Ca^2+^ handling is thought to contribute to the progression of HHD and may be a trigger for atrial tachyarrhythmias. [10].

LVH results in significant electrophysiological changes characterised by an increased electrical vulnerability on both cellular and tissue levels. These changes result in the dispersion of refractoriness and considerably lower ventricular fibrillation threshold. Moreover, an increased myocardial mass and interstitial myocardial fibrosis are associated with reduced coronary flow reserve [36]. Metabolic changes resulting from subendocardial ischemia, reduced metabolic tolerance of the hypertrophied myocardium to ischemia, low-grade inflammation, oxidative stress, and activation of the sympathoadrenergic system could facilitate proarrhythmic disturbances [142,143]. The prolongation of repolarisation due to increased LV mass, myocardial fibrosis, and myocardial ischemia may be associated with an increased risk of ventricular arrhythmias, including potentially malignant arrhythmias [36,142,143]. In addition to its impact on LV function, myocardial fibrosis (MF) provides a substrate for arrhythmogenicity and increases the risk of ventricular arrhythmias independently of LV function in HHD [144]. MF may induce conduction abnormalities, thereby promoting atrial and ventricular arrhythmia by creating a vulnerable substrate for re-entrant activity [145]. Patients with HHD and arrhythmias exhibit higher amounts of myocardial collagen than patients without arrhythmias, irrespectively of LVEF and the frequency of coronary artery disease, and ventricular arrhythmias are related to the extent of MF independently of other confounders, including LV dysfunction [102,145].

Animal studies have shown important arrhythmogenic effects of angiotensin II on the cardiac conduction system [138]. RAAS activation in HTN may be involved in the pathogenesis of arrhythmias including AF through several mechanisms. Angiotensin II promotes a direct arrhythmogenic effect by modulating ion channels in myocytes (reduced intracellular resistance, decreased conduction velocity, and shortened refractory period in cardiac myocytes) and through proinflammatory mechanisms [36,139,146,147]. Aldosterone, another component of RAAS, plays a key role in the development of AF with well-known effects on cardiac inflammation, fibrosis, first of all through upregulation of matrix metalloproteinases, hypertrophy, and possibly affecting ion channel function and distribution [139,147]. Also, both experimental and hypertensive human studies support the protective role of RAAS inhibition on the risk of AF [147].

Recently published studies suggested that atrial epicardial adipose tissue could upregulate the expression of some pro-inflammatory and pro-fibrotic adipokines while downregulating the expression of anti-fibrotic cytokines and predispose AF [148]. However, the role of epicardial adipose tissue in arrhythmias including AF in HHD is not yet fully understood.

#### 3.5.2. Hypertensive Heart Disease and Atrial Fibrillation

As the most often encountered arrhythmia, AF is associated with an increased risk of death, stroke/systemic embolism, and bleeding [138,149]. LVH and LA dilatation are the major predisposing conditions for the increased incidence of AF as the most often encountered arrhythmia in HHD [138]. ECG-diagnosed LVH in hypertensive patients has been shown to be associated with a 39% greater risk of new-onset AF [63,150]. A rise in LA stretch and pressure, with subsequent remodelling (LA dilatation, fibrosis) and dysfunction of the LA (so-called atrial cardiomyopathy) along with altered conduction velocity and heterogeneity ultimately predisposes AF and the perpetuation of AF while altered Ca^2+^ handling may serve as the trigger initiating the first episodes of AF [10,147]. From the other side, the presence of AF, even when the rate is controlled, causes LV remodelling with altered excitation–contraction coupling and ion homeostasis, and can impair LV function [10]. Moreover, the loss of the booster pump function in AF impairs LV function, which is one of the mechanisms by which AF can contribute to the high prevalence of HF in AF patients [10,151].

Generally, an increased likelihood for inducible atrial tachyarrhythmias has been shown for different animal models of HTN [10]. The role of HTN in the prediction, development, frequency, and maintenance of AF, the relation between different HHD patterns, and the incidence or AF in humans was shown in many randomized controlled trials (RCTs, e.g., LIFE, the Framingham study, the REGARDS (Reasons for Geographic and Racial Differences in Stroke) study, the Women’s Health Study) and meta-analyses [71,73,139,147]. Moreover, an 80% higher risk of AF in prehypertension compared to normotension and after multivariate adjustment for several potential confounders was confirmed in an analysis of the MESA study. Epidemiologic studies have shown that HTN is associated with a 1.8-fold increased risk of developing new-onset AF and a 1.5-fold increased risk of progression to permanent AF [139]. Verdecchia et al., in their study conducted on a large cohort of initially untreated hypertensive subjects, found that baseline LV hypertrophy almost doubled the risk of new-onset AF [72,147]. An association between BP levels and risk of new AF was shown in a group of 2014 healthy men in a Norwegian study with a median of 30 years follow-up [152]. However, though many clinical studies have shown a direct and linear relationship between BP levels and the risk of AF or the prevalence of AF in hypertensive patients, there are trials that do not support this relationship [147,153].

In patients with documented AF, hypertension increases the risk of thromboembolism and bleeding, which was confirmed in studies in anticoagulated patients with AF, and this facilitates the progression from paroxysmal to persistent or permanent AF [147,154]. Atrioventricular (AV) conduction disturbances, sinus node dysfunction (particularly sick sinus syndrome), morbidity, and mortality occur in hypertensive patients with persistent AF more than in normotensive patients [155].

#### 3.5.3. Other Arrhythmias and Conductions Disturbances and Hypertensive Heart Disease

Previous studies demonstrated that both SV and ventricular premature beats occur frequently in hypertensive patients with LVH [156]. In a recent meta-analysis of 10 studies including 27,141 patients, the incidence of SV tachycardias (AF or atrial flutter) in patients with LVH was 11.1% compared with 1.1% among those without LVH, and the incidence of VA was 5.5% compared with 1.2% in patients without LVH [71]. According to the international consensus document by the European Heart Rhythm Association (EHRA) and the ESC Council on Hypertension, endorsed by the Heart Rhythm Society (HRS), Asia-Pacific Heart Rhythm Society (APHRS), and Sociedad Latinoamericana de Estimulación Cardíaca y Electrofisiología (SOLEACE), patients with excessive SVPBs and LVH have a greater risk of developing AF, which is also associated with increased age, systolic BP, and NT-proBNP levels [156].

A subanalysis of the LIFE study found that the presence of a left bundle branch block (LBBB) significantly increased CV mortality (1.6-fold more), risk of sudden cardiac death (3.5-fold more), and symptomatic HF (1.7-fold more) in patients with hypertension and LVH compared with those without LBBB [157].

The intensive studying of the relationship between AF and HTN in recent decades has provided valuable information to many questions, but still aspects from a clinical point of view remain not fully elucidated. Therefore, it is not clear whether the risk of AF increases linearly with BP level or whether there is a certain BP threshold above which the risk of this arrhythmia increases. Additionally, the role of out-of-office BP and the cumulative BP load as risk predictors in the setting of HHD and AF, as well as arrhythmia risk, have not been entirely studied. Further, it is not clear if the use of specific antihypertensive and especially antifibrotic drugs reduces AF burden or the risk of new-onset AF in patients with sinus rhythm. Moreover, the ideal BP level for hypertensive patients with anticoagulated AF to minimize the risk for haemorrhagic and thrombolytic complications is uncertain. Also, the role of novel biomarkers of structural or functional cardiac changes as risk predictors of arrhythmias, including AF, remains to be clarified. As the risk of arrhythmia is closely related to the structure and remodelling of the heart, the role of new imaging techniques needs also to be determined, as well as when they should be used, to properly identify patients with high arrhythmogenic risk in HHD patients.

## 4. Conclusions and Perspectives

This review attempts to analyse the constellation of the currently available experimental and clinical data on macroscopic structural changes, including left and right heart alterations, heart failure, and rhythm and conduction disturbances as clinical manifestations of developing and progressing HHD.

Thus, over the last decade, a number of extensive research works addressed the complexity of the causal relationships between structural and as well as functional alterations in HHD. The application of modern technology and methodology has resulted in major advances in our understanding of the pathophysiological processes occurring in HHD and its consequences. There is strong evidence for the influence of chronic high blood pressure on changes that occur in the left heart, such LVH and fibrosis, as well as LA remodelling, with their consequences of HF and arrhythmias. However, significant gaps remain in the literature regarding the impact of HTN on the right heart chambers and their involvement and role in HHD progression. It is worth noting that the mechanisms linking LVH to the risk of major CV events as well as the mechanisms by which regression of LVH reduces the risk of CV disease in hypertensive subjects are also still incompletely understood. The correct evaluation of the heterogenous cardiac alterations occurring in HHD is important because they have been established as strong predictors of fatal and non-fatal adverse clinical outcomes in both asymptomatic hypertensive individuals and those with prevalent disease.

With the predicted increase in the burden of hypertension and life expectancy over the next few decades, HHD can be expected to have an even greater impact in the future of health care. Further studies are needed to elucidate the relative prognostic contributions of distinct singular or combined phenotypes that constitute HHD and create the optimal approach to stratify and reduce CV in different patterns of HHD.

## Figures and Tables

**Table 1:** Geometric patterns of LV in hypertensive patients.

Relative Wall Thickness	>0.42	Concentric remodelling 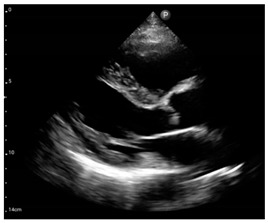	Concentric hypertrophy 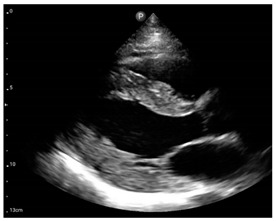
	≤0.42	Normal geometry 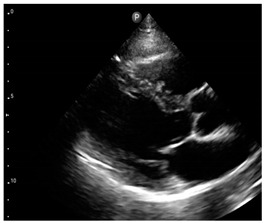	Eccentric hypertrophy 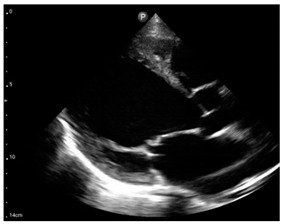
		m ≤ 115 f ≤ 95	m > 115 f > 95
		Left Ventricular Mass Index (g/m^2^)	

**Table 2:** A 4-group classification of left ventricular hypertrophy.

LVH Subgroups (by CMR)	LVH Subgroups (by Echo)	LV Mass	LV EDV	RWT
Indeterminate LVH	Eccentric non-dilated	+	-	-
Dilated LVH	Eccentric dilated	+	+	-
Thick LVH	Concentric non-dilated	+	-	+
Both thick and dilated LVH	Concentric dilated	+	+	+

Note: LV mass—left ventricular mass: “-”—for males ≤ 115 gm/m^2^, for females ≤ 95 gm/m^2^, “+”—for males > 115 gm/m^2^, for females > 95 gm/m^2^; LV EDV—left ventricular end-diastolic volume: “-”—EDV ≤ 75 mL/m^2^, “+”—>75 mL/m^2^; RWT—relative wall thickness: “-”—RWT ≤ 0.42, “+”—RWT > 0.42.

## Data Availability

Not applicable.
